# Author Correction: Restoration of patterned vision with an engineered photoactivatable G protein-coupled receptor

**DOI:** 10.1038/s41467-018-03585-2

**Published:** 2018-03-13

**Authors:** Michael H. Berry, Amy Holt, Joshua Levitz, Johannes Broichhagen, Benjamin M. Gaub, Meike Visel, Cherise Stanley, Krishan Aghi, Yang Joon Kim, Kevin Cao, Richard H Kramer, Dirk Trauner, John Flannery, Ehud Y. Isacoff

**Affiliations:** 1 Department of Molecular and Cell Biology, University of California, Berkeley, CA 94720 USA; 2 Department of Medicine, Oregon Health and Science University, Portland, OR 97239 USA; 3 Department of Chemistry, Ludwig-Maximilians-Universität München and Munich Center for Integrated Protein Science, Butenandtstrasse 5-13, 81377 München, Germany; 4 Laboratory of Protein Engineering, Institut des Sciences et Ingénierie Chimiques, Sciences de Base, École Polytechnique Fédérale Lausanne, 1015 Lausanne, Switzerland; 5 Department of Biosystems Science Engineering, ETH Zürich, Mattenstrasse 26, 4058 Basel, Switzerland; 6 Helen Wills Neuroscience Institute, University of California, Berkeley, CA 94720 USA; 7 Biophysics Graduate Program, University of California, Berkeley, CA 94720 USA; 8 School of Optometry, University of California, Berkeley, CA 94720 USA; 9 Bioscience Division, Lawrence Berkeley National Laboratory, Berkeley, CA 94720 USA; 10 Present Address: Department of Biochemistry, Weill Cornell Medical College, New York City, New York 10024 USA; 11 Present Address: Department of Chemical Biology, Max-Planck Institute for Medical Research, Jahnstr. 29, 69120 Heidelberg, Germany; 12 Present Address: Department of Chemistry, New York University, New York City, New York 10003 USA

*Nature Communications* **8**:1862 10.1038/s41467-017-01990-7; Article published online: 30 Nov 2017

Kevin J. Cao and Richard H. Kramer, who developed extended release with beta cyclodextrin, were inadvertently omitted from the author list and author contributions section of this Article. These errors have now been corrected in both the PDF and HTML versions of the Article.

