# Supplementary material for: Restoration of patterned vision with an engineered photoactivatable G protein-coupled receptor
Source: Nat Commun. 2017 Nov 30;8:1862. doi: 10.1038/s41467-017-01990-7 (PMC5709376; doi:10.1038/s41467-017-01990-7)
Supplement: Supplementary file 1 — Supplementary Information [file 41467_2017_1990_MOESM1_ESM.pdf]

Supplementary Note 1:

Characterization of SNAG-mGluR2 in HEK293 cells:

Photo-isomerization of the azobenzene moiety from *trans* to *cis* enables glutamate binding and activates the receptor (Supplementary Fig. 1a). Two BGAG variants were tested: i) a bi-stable $BGAG_n$ family that is activated (isomerized to *cis*) by 380 nm light and deactivated (isomerized to *trans*) by 500 nm light, and ii) a red-shifted spontaneously-relaxing $BGAG_{n,460}$ family that is activated by visible light (peak efficiency at 460 nm) and spontaneously turns off in the dark [1]. Expression of SNAP-mGluR2 in HEK293 cells co-expressing the G protein-coupled inward-rectifier potassium (GIRK) channel followed by labeling with $BGAG_{12,460}$ yielded robust inward photocurrent that turned off when the light was extinguished (Supplementary Fig. 1b), unlike currents observed in cells labeled with $BGAG_{12}$ which showed similar kinetics but required illumination with the longer wavelength of light to turn off (Supplementary Fig. 1b). The one-wavelength $BGAG_{12,460}$ had a photocurrent amplitude that was light-intensity dependent over three orders of magnitude (Supplementary Fig. 1c,d) and responded to light pulses (at 1 mW mm^{-2}) as short as 50 ms (Supplementary Fig. 1e,f). The intensity-dependence and speed of SNAG-mGluR2 with $BGAG_{12,460}$ display desirable characteristics suggesting suitability for vision restoration.

Supplementary Figures & Legends

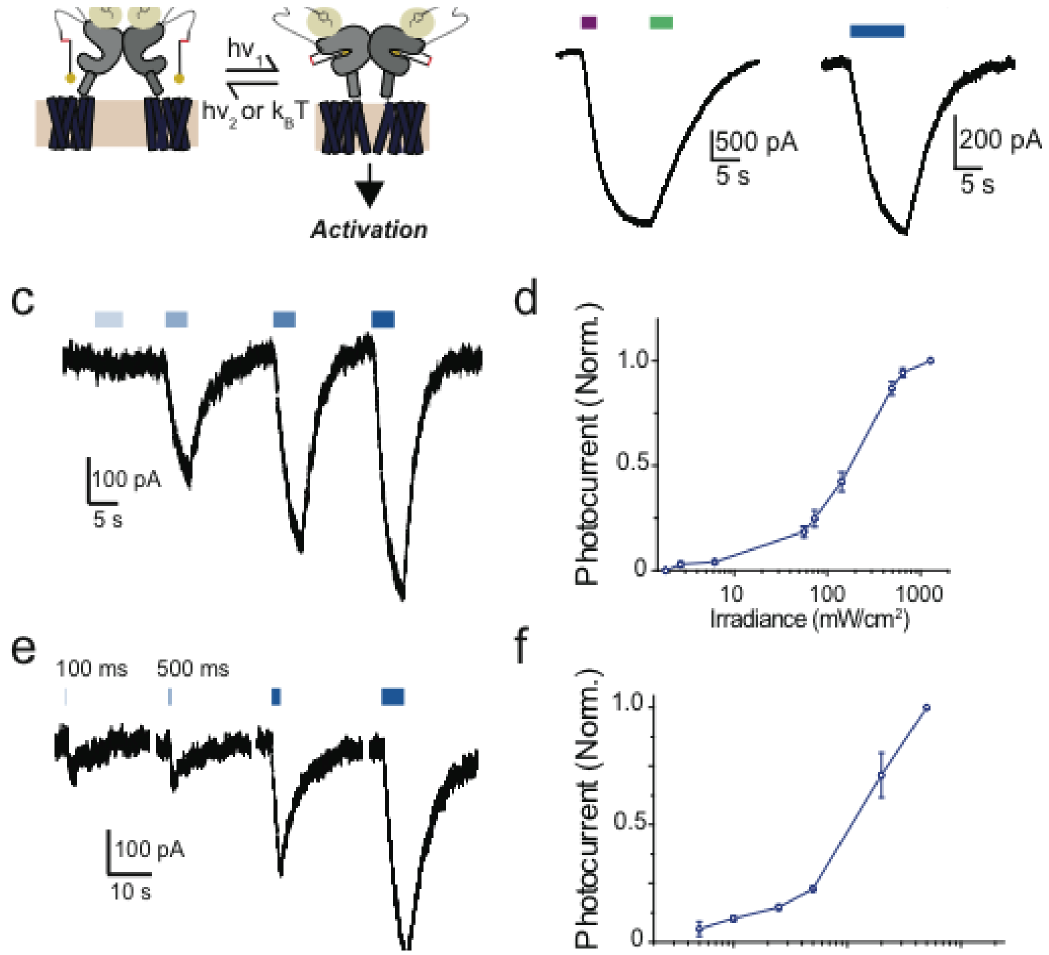

Supplementary Figure 1. Optical control of SNAG-mGluR2 in HEK293 cells.

(a) Cartoon of SNAP-mGluR2 with BGAG covalently bound *via* BG moiety to the SNAP tag. Inactive (*trans*, left) and active (*cis*, right) states shown in response to different illumination conditions.

(b-d) Light control of SNAP-mGluR2 by light detected in whole cell patch clamp *via* activation of co-expressed GIRK channels.

(b) (Left) Optical control with $BGAG_{12}$: activating *cis* isomerization by 380 nm light (violet) and deactivating *trans* isomerization by 500 nm light (green) (Note that the activated state is stable in then non-illuminated interval). (Right) Optical control with $BGAG_{12,460}$ ("SNAG-mGluR2"): activating *cis* isomerization by 445 nm light and spontaneous deactivation in the dark.

(c,d) Irradiance dependence of SNAG-mGluR2 photoactivation. Representative patch-clamp trace (c) and normalized photocurrent amplitudes (mean $\pm$ SEM.; n=5 cells) (d).

(e,f) Dependence on light pulse duration of SNAG-mGluR2 photoactivation. (e) Representative patch-clamp trace (e) and normalized photocurrent amplitudes (mean $\pm$ SEM; n=6 cells) (f).

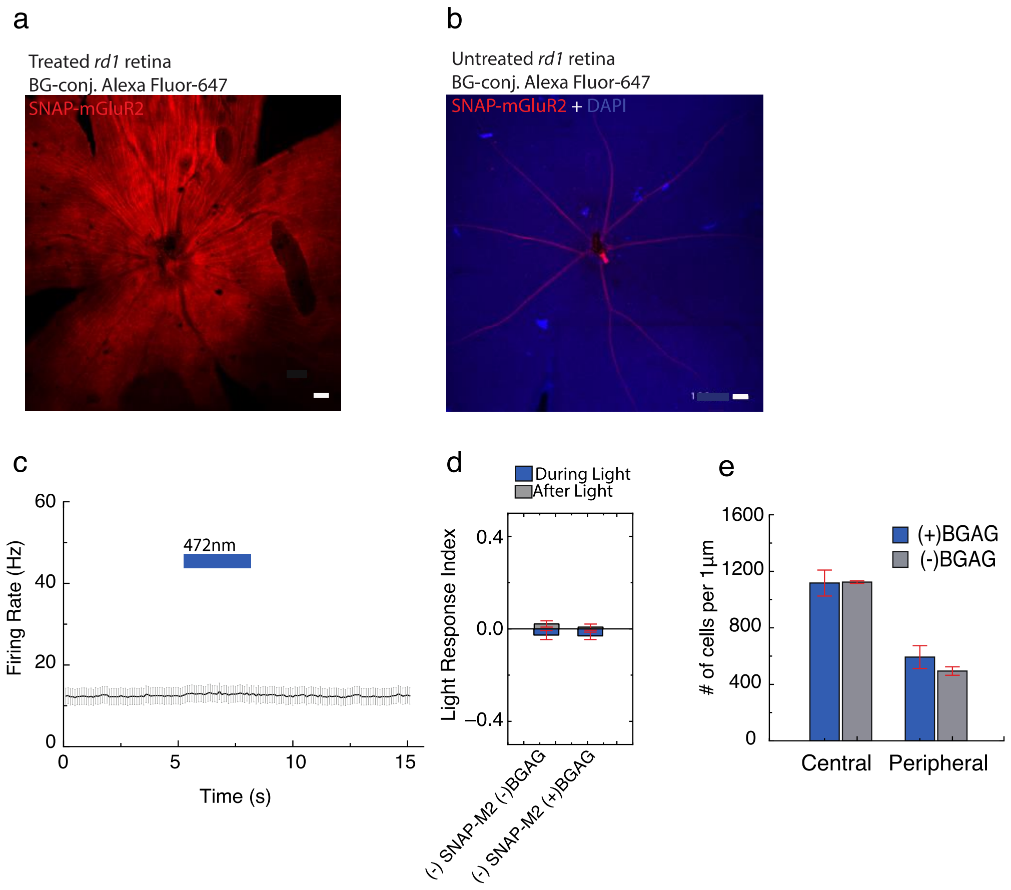

Supplementary Figure 2. Selectivity and safety of SNAP-mGluR2 expression in *rd1* retina

(a,b) Flat mount confocal images of *rd1* retina that received (a) (treated) or did not receive (b) (untreated) intravitreal injection of *AAV2/2-hSyn-SNAP-mGluR* (2 µL volume containing 5 x $10^{10\text{-}11}$ viral genomes). *SNAP*-Surface *Alexa* Fluor *647* dye (red) used to visualize SNAP-mGluR2 and DAPI (blue) to visualize nuclei. Scale of 100 µm.

(c) Average response of RGC population in (SEM in gray) to 3 s light illumination from *rd1* mouse retina not expressing SNAP-mGluR2 but exposed to $BGAG_{12,460}$ (n=50) (λ= 445 nm).

(d) Normalized Light Response Index (LRI = peak ON [blue] or OFF [grey] firing rate - average firing rate in dark) for retina not expressing SNAP-mGluR2 before (Left) and after (Right) the addition of $BGAG_{12,460}$.

(e) Retinas from rd1 mice expressing SNAP-mGluR2 show no effect on cell density in central or peripheral areas 6 months after repeat intravitreal injections (2x) of BGAG12,460 at 1 mM (equal to 500 times the effective dose). N = 2 retina per condition.

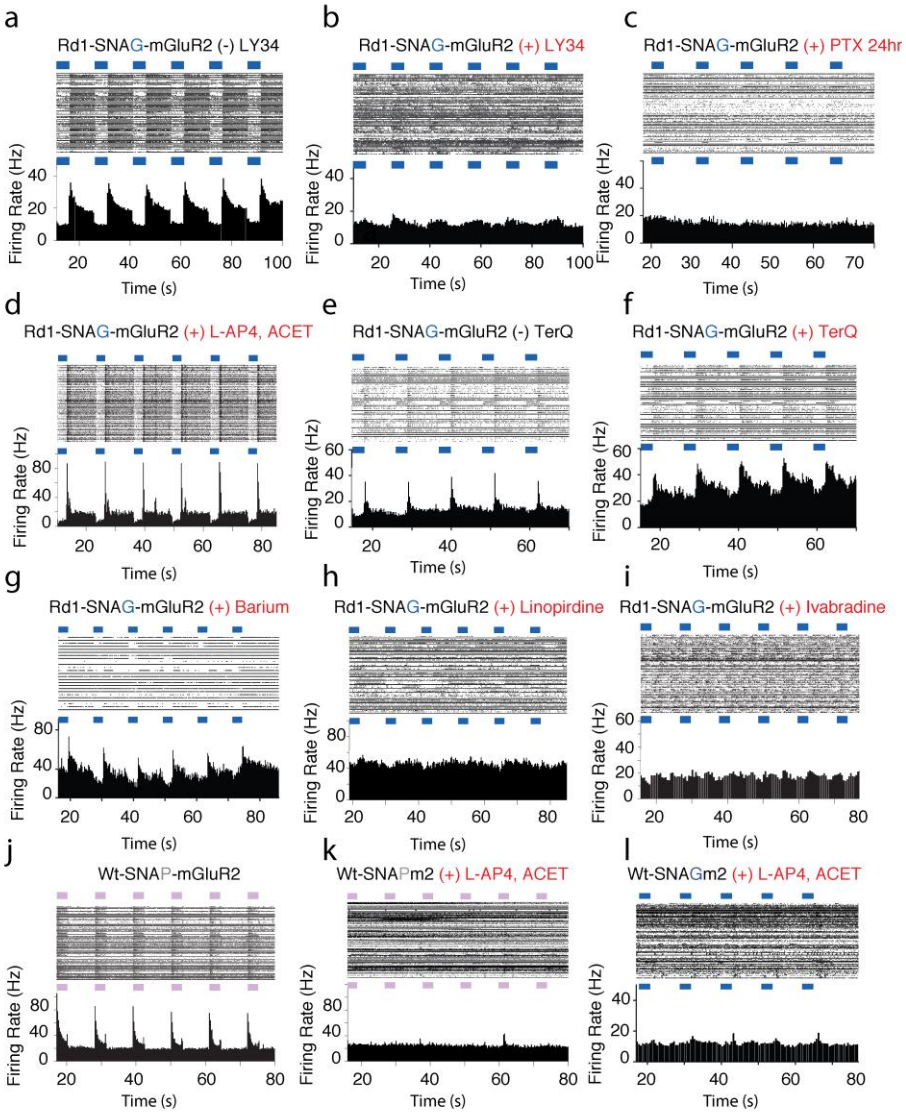

Supplementary Figure 3: Representative recordings of *rd1* or *wt* retina under pharmacological exposure

(a-b) Representative MEA recordings from *rd1* mouse retina expressing SNAG-mGluR2 in RGCs before (a) and after (b) addition of 5 µM LY341495 (a,b: n=71). (c) Representative MEA recordings from *rd1* mouse retina expressing SNAG-mGluR2 in RGCs following retinal injected with 150 uM pertussis toxin (PTX) for 24 hrs (c: n=60).

(d-i) Representative MEA recordings from *rd1* mouse retina expressing SNAG-mGluR2 in RGCs in the presence of 50µM L-AP4 & 1µM ACET (d), 300 nM Tertiapin-Q (e=before, f=after), 1mM barium (g), 500 nM linopirdine (h), or 50 µM ivabradine (i) in the recording solution (d,e,f,g,h,i: n=121,50,50,31,67,47).

(j-k) Representative MEA recordings from photoreceptor intact *wt* mouse retina expressing SNAP-mGluR2 in RGCs before (j) and after (k) 50µM L-AP4 & 1µM ACET and $BGAG_{12,460}$ under white light (k) or 445nm light stimulation (l) (j,k,l: n=80). (Top) Raster plot with spikes for each RGC identified on the MEA. (Bottom) Peristimulus time histogram (PSTH). Light stimulation protocol: 5 x 3 s of light (λ= 445 nm, blue bars or white light, pink bars) separated by 10 s dark.

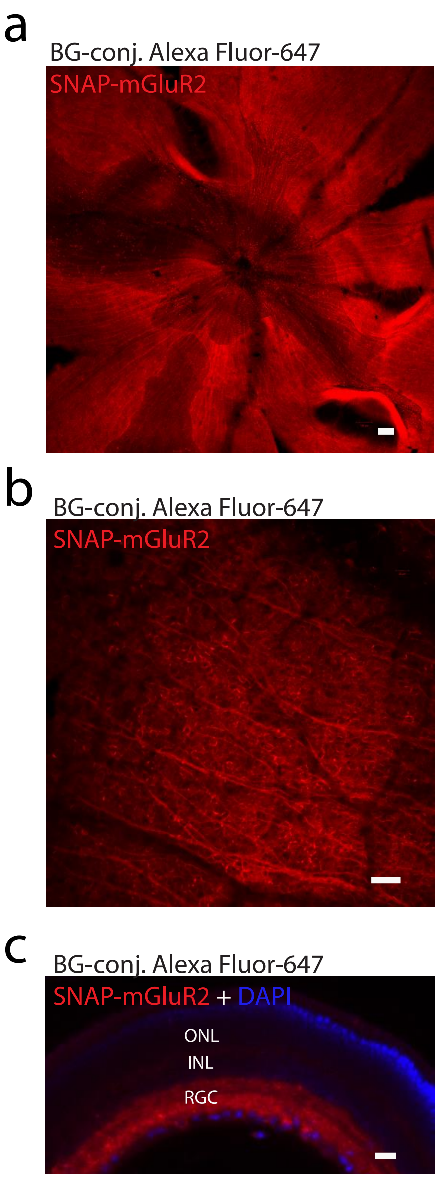

Supplementary Figure 4: SNAP-MGluR2 expresses in *wt* retina

(a,b,c) Flat mount (a) and slice (b,c) confocal images of SNAP-mGluR2 expression in RGCs of *wt* mouse retina 4 wks after intravitreal injection of AAV2/2-hSyn-SNAP-mGluR (2 μL volume containing 5 x $10^{10\text{-}11}$ viral genomes). *SNAP*-Surface *Alexa* Fluor *647* dye (red) used to visualize SNAP-mGluR2 and DAPI (blue) to visualize nuclei. Scale bar of 100 (a), 40 (b) & 20 (c) μm.

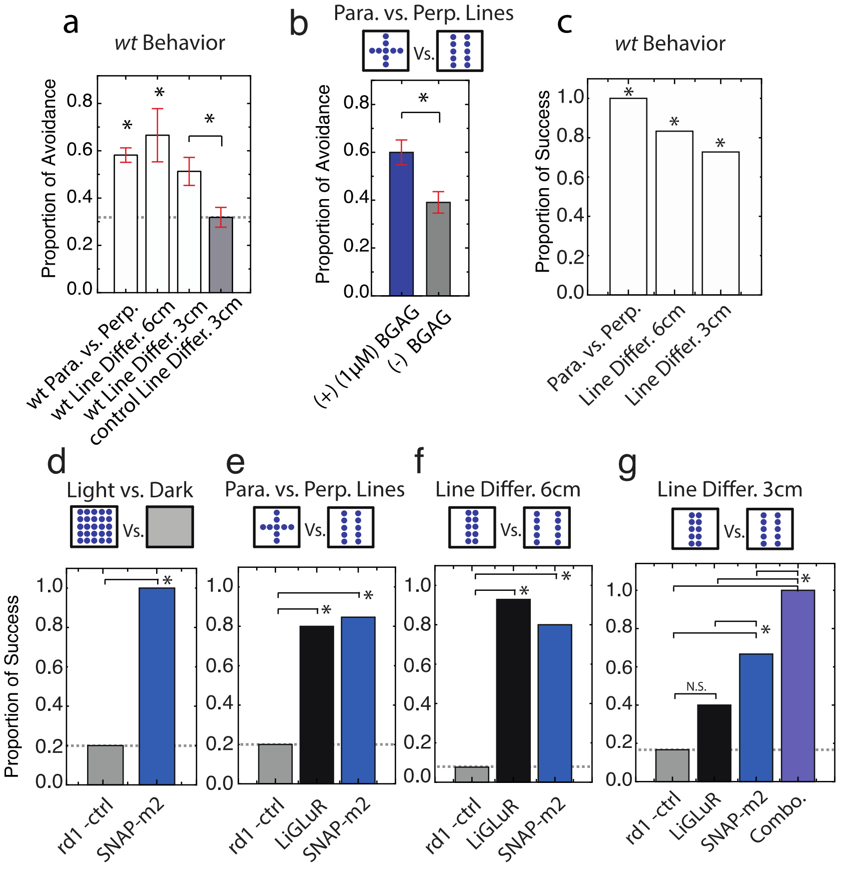

Supplementary Figure 5: Learned pattern discrimination behavior

(a) Proportion of time spent avoiding pattern paired with shock for *wt* mice. Successful discrimination of perpendicular vs. parallel bars. (white-Left), parallel bars at distances of 1 cm vs. 6 cm (white-middle) or 1 cm vs. 3 cm (white-right). The performance of untreated *rd1* mice (control) in line differentiation of 1 cm vs. 3 cm is replicated from Fig. 4h as a point of comparison. Respectively, left, middle, and right wildtype n=6, 6 and 11 and control *rd1* (right-gray) n=12.

(b) Proportion of time spent avoiding pattern paired with shock following injection with 500x lower BGAG concentration (final vitreal concentration of 1 µM). Successful discrimination of perpendicular vs. parallel bars compared to same mice 3 weeks following injection (-) BGAG. Student's paired two-tailed t test * = $p < 0.05$

(c) Proportion of successful avoidance of pattern paired with shock for *wt* mice. Successful discrimination of perpendicular vs. parallel bars. (Left), parallel bars at distances of 1 cm vs. 6 cm (middle) or 1 cm vs. 3 cm (right). Respectively, left, middle, and right *wt* n=6, 6 and 11.

(d) Learned dark avoidance behavior. Proportion of successful avoidance of dark after a conditioning period where dark was paired with a shock. *rd1* SNAG-mGluR2 (n=6), *rd1* control (n=6).

(e-g) Learned pattern discrimination. Proportion of successful avoidance of patterns paired with shock. (e) Perpendicular vs. parallel bars. (f, g) Discrimination of parallel bars at distances of 1 vs. 6 cm (f) or 1 vs. 3 cm (g). Respectively for e,f, and g: *rd1* control (n=7,13,12 mice), *rd1* LiGluR (n=7,14,15 mice), *rd1* SNAG-mGluR2

(n=7,10,18 mice), rd1 combo. (n=18). Statistical significance was assessed using Student's two-tailed t test *p < 0.05 (a,c) and Two-Sided Pearson's Chi-Square Test and One-Sided Fisher's Exact Test when applicable (b,d-g) (See Supplementary Table 3)

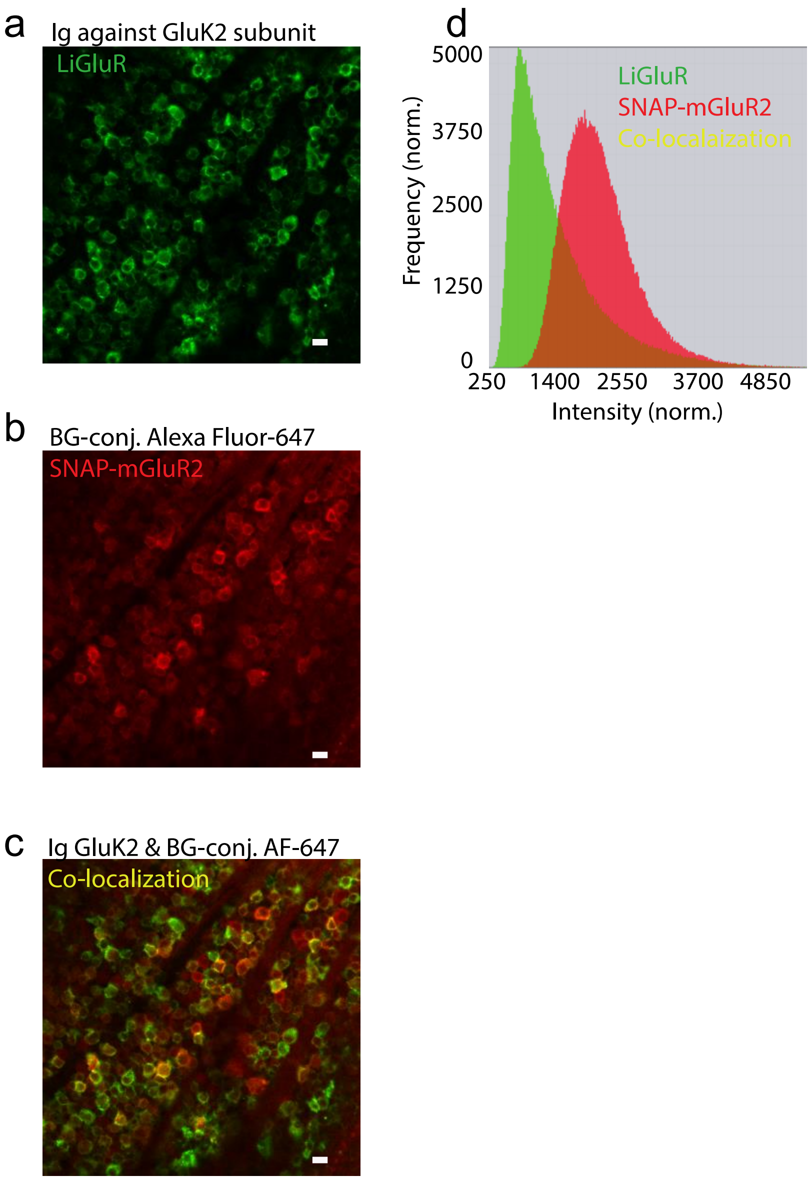

Supplementary Figure 6: Combined expression of SNAP-mGluR2 and LiGluR yields a range of expression levels across the RGC population.

(a-d) Confocal images of LiGluR stained with anti-iglur6/7 antibody (green) (a) and SNAP-mGluR2 stained with BG-conjugated Alexa Fluor 647 (red) (b) and their co-localization (c) and relative intensity (d) in RGCs of *rd1* mouse retina > 4wks after intravitreal injection of mixture 1:1 of AAV2/2-hSyn-LiGluR and AV2/2-hSyn-SNAP-mGluR2 (2-µl volume equal to 5 x 10^{11} viral genomes each) Scale bar of 20 µm.

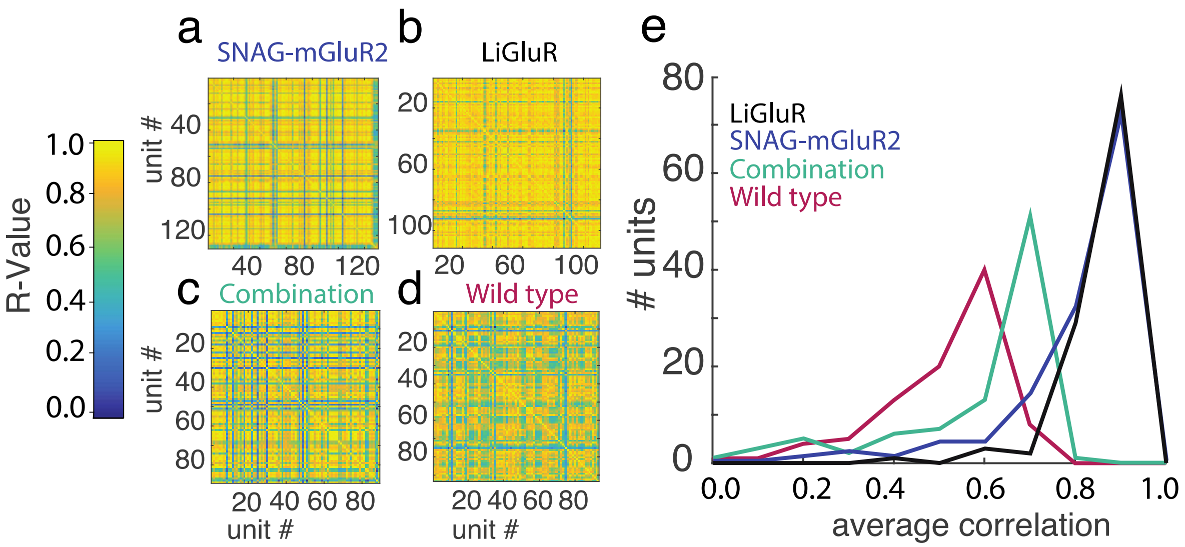

Supplementary Figure 7: Correlation matrices showing degree of similarity between RGC light responses measured on the MEA.

(a-d) Correlation matrices showing correlations between all light-sensitive single units from within the same retina in *rd1* retinas expressing SNAG-mGluR2 alone (a), LiGluR alone (b), SNAG-mGluR2 and LiGluR together (c) and wildtype retina (d). RGC responses during 1 s before through 2 s after illumination were used for the correlation. The color of the heat map indicates the magnitude of the correlation value r, with warmer colors indicating higher values. (Number of units per retina: a, n=134; b, n=120; c, n=90; d, n=93). **(e)** Correlative value distributions for light sensitive units within the same retina for SNAG-mGluR2 alone (blue), LiGluR alone (black), co-expressed SNAG-mGluR2 + LiGluR (green), and wildtype (red) reveals that co-expression of SNAG-mGluR2 + LiGluR approaches the diversity of the wildtype retina.

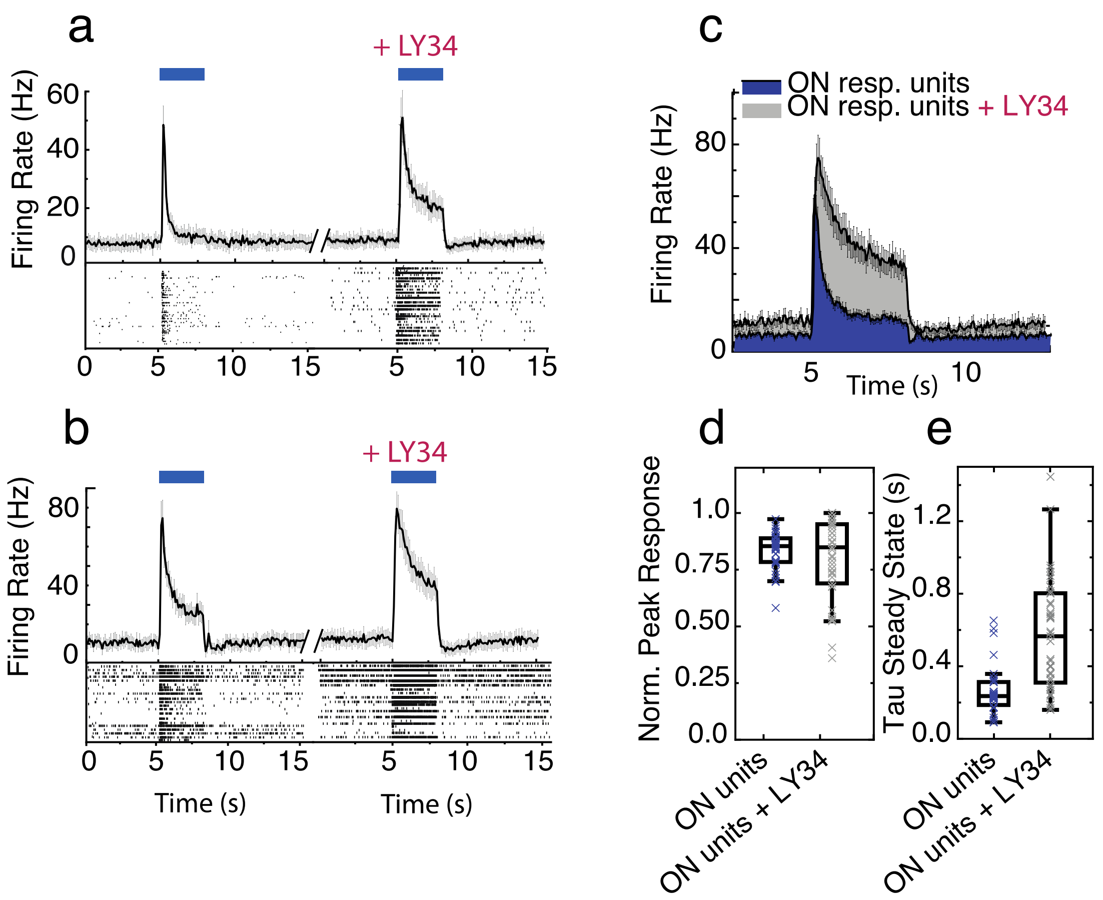

Supplementary Figure 8: Light response diversity due to combined activity of SNAG-mGluR2 and LiGluR

(a,b) Average response (top) and raster (bottom) of ON transient (a) and ON sustained (b) units from a representative retina co-expressing SNAG-mGluR2 and LiGluR before and after addition of the competitive mGluR2 antagonist 5 μM LY341495. (c) Combined response to 3 s light for all ON responsive units from co-expressing retina before (blue) and after (gray) antagonist. (d) Normalized peak light response index shows no effect on peak light response of LY341495. (e) Decay kinetics of ON responsive cells before (Tau $_{ONdecay}$ = 0.260 ± 0.022) (blue) and after (Tau $_{ONdecay}$ 0.531 ± 0.036) (gray) LY341495 (n=69). Statistical significance was assessed using Mann-Whitney U test (*p<0.001) (See Supplementary Table 1).

Mann-Whitney U test (two tailed)

		Significance?
***Rd1* SNAP-mGluR2**		
**BGAG (Fig. 1h)**		
(-) BGAG inhibition vs (+) BGAG inhibition	p<0.001	Yes
(-) BGAG OFF response vs (+) BGAG OFF response	p<0.001	Yes
***Rd1* SNAG-mGluR2**		
**LY34 (Fig. 1h)**		
(-) drug inhibition vs (+) drug inhibition	p<0.001	Yes
(-) drug OFF response vs (+) drug OFF response	p<0.001	Yes
**PTX (Fig. 1h)**		
(-) drug inhibition vs (+) drug inhibition	p<0.001	Yes
(-) drug OFF response vs (+) drug OFF response	p<0.001	Yes
**Liprodine (Fig. 1i)**		
(-) drug inhibition vs (+) drug inhibition	p=0.048	Yes
(-) drug OFF response vs (+) drug OFF response	p<0.001	Yes
**Ivibradine (Fig. 1i)**		
(-) drug inhibition vs (+) drug inhibition	p = 0.001	Yes
(-) drug OFF response vs (+) drug OFF response	p<0.001	Yes
**wt SNAG-mGluR2 response (20mW/cm2) (Fig. 1k)**		
wt SNAG-mGluR2 inhibition vs *rd1* SNAGmGluR2 inhibition	p<0.001	Yes
wt SNAG-mGluR2 OFF vs *rd1* SNAG-mGluR2 OFF	p<0.001	Yes
**wt SNAG-mGluR2 response (50mW/cm2) (Fig. 1k)**		
wt SNAG-mGluR2 inhibition vs rd1 SNAG-mGluR2 inhibition	p<0.001	Yes
wt SNAG-mGluR2 OFF vs rd1 SNAG-mGluR2 OFF	p<0.001	Yes
**Concentration Dep 500 nM (Fig. 2c)**		
in vivo inhibition vs in vitro inhibition	p <0.001	Yes
in vivo OFF vs in vitro OFF	p <0.001	Yes
**cross correlation (Fig. 4k)**		
Rd1 SNAG-mGLuR2 vs *wt* c57	p<0.001	Yes
Rd1 SNAG-mGLuR2 vs *rd1* combo.	p<0.001	Yes
Rd1 LiGluR vs *wt* c57	p<0.001	Yes
Rd1 LiGluR vs *rd1* combo.	p<0.001	Yes
**Combo. Peak response (Fig. S8d)**		
(-) LY34 vs (+) LY34	p = 0.674	No
**Combo. tau OFF (Fig. S8e)**		
(-) LY34 vs (+) LY34	p<0.001	Yes
cross correlation (Fig. 4k)		
Rd1 SNAG-mGLuR2 vs wt c57	p<0.001	Yes
Rd1 SNAG-mGLuR2 vs rd1 combo.	p<0.001	Yes
Rd1 LiGluR vs wt c57	p<0.001	Yes
Rd1 LiGluR vs rd1 combo.	p<0.001	Yes
**cross correlation (Fig. 4k)**		
Rd1 SNAG-mGLuR2 vs *wt* c57	p<0.001	Yes
Rd1 SNAG-mGLuR2 vs *rd1* combo.	p<0.001	Yes
Rd1 LiGluR vs *wt* c57	p<0.001	Yes
Rd1 LiGluR vs *rd1* combo.	p<0.001	Yes

Supplementary Table 1: Statistical significance of pertinent MEA recordings

Statistical significance for RGC response was assessed using Mann-Whitney U test. (See figure legends for details)

**repeated measure *ANOVA***			
(days 7-42)	sphericity assumed	Greenhouse-Geisser correction	
Rd1 SNAP-mGluR2 with cyclodextrin + BGAG	p = 0.418	p = 0.401	Statistically insignificant
Rd1 untreated control	p = 0.622	p = 0.510	Statistically insignificant
**Between subject one-way *ANOVA***			
Rd1 SNAP-mGluR2 with cyclodextrin + BGAG vs Rd1 untreated control		p < 0.005 for days 7-42	Statistically significant

Supplementary Table 2: Statistical analysis of behavioral light response following single injection of slow release BGAG.

Statistical significance calculations for slow release BGAG delivery for behavioral light avoidance (Fig. 3b) analyzed by repeated-measures ANOVA (rANOVA) using "time point" as the within-subject variable and "group" (control, treated) as the between-subject variable. Within-subject effects were analyzed by one-way ANOVA using "time point" as the independent variable. Where sphericity was violated, as assessed by Maulchy's test of sphericity, the Greenhouse–Geisser correction was applied.

**Dark averse (Fig. S5d & Fig. 3e)**	Pearson's Chi-Square Test (2 sided)	Fisher's Exact Test (1 sided)	Significance?
Rd1 SNAG-mGluR2 vs rd1 control	0.008	0.003	Yes

**Perp. vs. parallel bars (Fig. S5e & Fig. 3f)**	Pearson's Chi-Square Test (2 sided)	Fisher's Exact Test (1 sided)	Significance?
Rd1 SNAG-mGluR2 vs *rd1* LiGluR	0.814	0.65	No
Rd1 SNAG-mGluR2 vs *rd1* untreated control	0.002	0.004	Yes
Rd1 SNAG-mGluR2 vs *wt* c57	0.352	0.51	No
Rd1 LiGluR vs *rd1* untreated control	0.023	0.045	Yes
Rd1 LiGluR vs *wt* c57	0.292	0.5	No
Rd1 untreated control vs *wt* c57	0.003	0.008	Yes

**Line differentiation (Fig. S5f Fig. 3g)**	Pearson's Chi-Square Test (2 sided)	Fisher's Exact Test (1 sided)	Significance?
Rd1 SNAG-mGluR2 vs *rd1* LiGluR	0.814	0.371	No
Rd1 SNAG-mGluR2 vs *rd1* untreated control	0.002	0.001	Yes
Rd1 SNAG-mGluR2 vs *wt* c57	0.352	0.489	No
Rd1 LiGluR vs *rd1* untreated control	0.023	<0.001	Yes
Rd1 LiGluR vs *wt* c57	0.292	0.202	No
Rd1 untreated control vs *wt* c57	0.003	0.017	Yes

**Close line differentiation (Fig. S5g,h Fig. 3h & 5i)**	Pearson's Chi-Square test (2 sided)	Fisher's Exact Test (1 sided)	Significance?
Rd1 SNAG-mGluR2 vs *rd1* untreated control	0.025	0.03	Yes
Rd1 SNAG-mGluR2 vs *wt* c57	0.732	0.534	No
Rd1 LiGluR vs *rd1* untreated control	0.411	0.343	No
Rd1 LiGluR vs *wt* c57	0.098	0.104	No
Rd1 untreated control vs *wt* c57	0.022	0.03	Yes
Rd1 SNAG-mGluR2 vs *rd1* combo.	0.007	0.01	Yes
Rd1 LiGluR vs *rd1* combo.	<0.001	<0.001	Yes
Rd1 untreated control vs *rd1* combo.	<0.001	<0.001	Yes
wt c57 vs *rd1* combo.	0.019	0.045	Yes

Supplementary Table 3: Statistical significance of learned discrimination behaviors

Success ratios were calculated for avoidance performance of condition behaviors (Fig. 3e-h & Supplementary Fig. 5b,d-g). To determine significance in differences between conditions a pairwise contingency table was then constructed, and a Two-Sided Pearson's Chi-Square Test was initially conducted. To correct for conditions with a small n, a One-Sided Fisher's Exact Test was also conducted.

Supplementary References

1. Broichhagen, J., *et al.* Orthogonal Optical Control of a G Protein-Coupled Receptor with a SNAP-Tethered Photochromic Ligand. *ACS Cent Sci* **1**, 383-393 (2015).
